# Activation of TCA cycle restrains virus-metabolic hijacking and viral replication in mouse hepatitis virus-infected cells

**DOI:** 10.1186/s13578-021-00740-z

**Published:** 2022-01-18

**Authors:** Sang R. Lee, Jeong Yeon Roh, Jihoon Ryu, Hyun-Jin Shin, Eui-Ju Hong

**Affiliations:** College of Veterinary Medicine, Chungnam National University, 99 Daehak-ro, Yuseong-gu, Daejeon, 34134 Republic of Korea

**Keywords:** MHV, Coronavirus, Mitochondria, TCA cycle, Glycolysis, Metabolism, Pyruvate, Fatty acids, Glutamine, NR

## Abstract

**Background:**

One of coronavirus, severe acute respiratory syndrome coronavirus 2 (SARS-CoV-2), has caused coronavirus disease 2019 (COVID-19) pandemic and threatened worldwide. However, therapy for COVID-19 has rarely been proven to possess specific efficacy. As the virus relies on host metabolism for its survival, several studies have reported metabolic intervention by SARS-CoV-2.

**Results:**

We investigated the coronavirus-metabolic hijacking using mouse hepatitis virus (MHV) as a surrogate for SARS-CoV-2. Based on the altered host metabolism by MHV infection, an increase of glycolysis with low mitochondrial metabolism, we tried to investigate possible therapeutic molecules which increase the TCA cycle. Endogenous metabolites and metabolic regulators were introduced to restrain viral replication by metabolic intervention. We observed that cells deprived of cellular energy nutrition with low glycolysis strongly suppress viral replication. Furthermore, viral replication was also significantly suppressed by electron transport chain inhibitors which exhaust cellular energy. Apart from glycolysis and ETC, pyruvate supplement suppressed viral replication by the TCA cycle induction. As the non-glucose metabolite, fatty acids supplement decreased viral replication via the TCA cycle. Additionally, as a highly possible therapeutic metabolite, nicotinamide riboside (NR) supplement, which activates the TCA cycle by supplying NAD+, substantially suppressed viral replication.

**Conclusions:**

This study suggests that metabolite-mediated TCA cycle activation suppresses replication of coronavirus and suggests that NR might play a role as a novel therapeutic metabolite for coronavirus.

**Supplementary Information:**

The online version contains supplementary material available at 10.1186/s13578-021-00740-z.

## Background

Betacoronavirus is one of four genera of coronavirus and consists of four lineages including Embecovirus, Sarbecovirus, Merbecovirus, and Nobecovirus. In lineage B, the Sarbecovirus contains a Severe acute respiratory syndrome coronavirus 2 (SARS-CoV-2). SARS-CoV-2 induced coronavirus disease 2019 (COVID-19) was defined as a pandemic by World Health Organization (WHO), and the number of death by COVID-19 exceeded 1 million people on October, 2020 [1]. While the importance and urgency for treating COVID-19 are evident, there is few specific therapeutic agent for rescuing from SARS-CoV-2 though molecular and experimental approaches were vigorously discussed [2]. Remdesivir, which is RNA-dependent RNA polymerase (RDRP) inhibitor, shortened rehabilitation of COVID-19 patients in randomized clinical test [3], however, did not improve clinical status in another randomized clinical trial [4]. Other treatments, lopinavir-ritonavir [5] and chloroquine-hydroxychloroquine [6], also failed to present satisfying results due to retarded improvement or lacking methodology. While developing therapeutic agents had been in difficulty, diversely designed vaccines for SARS-CoV-2 have been introduced and obtained efficacy [7, 8]. However, as prevalence of SARS-CoV-2 mutants is expanding, there is still an urgent need to find an effective therapeutic approach for COVID-19 patients.

A virus is an intracellular obligate parasite relying on host cells and reprograms host metabolism for its replication [9]. Among RNA viruses, the dengue virus induces glucose uptake and glycolysis [10], and the zika virus increases glycolysis for activating pentose phosphate pathway [11]. Importantly, SARS-CoV-2 increases glycolysis in monocyte; their replication in monocyte was suppressed by hexokinase inhibitor, 2-deoxyglucose [12]. Furthermore, SARS-CoV-2 has been known to induce and use the Warburg effect for their replication [13]. Meanwhile, SARS-CoV-2 decreases TCA cycle activation and oxidative phosphorylation, contributing to systemic toxicity and lethality of patients [14, 15]. Herein, we tried to investigate metabolic regulators involved in viral suppression, which can be used as therapeutic agents.

To investigate the host metabolic alteration of SARS-CoV-2, we studied mouse hepatitis virus (MHV; JHM-cl2) as a surrogate. While HCoV-OC43 is a closer mimetic of SARS-CoV-2 than MHV, the MHV has been used for animal disease models in many virological and clinical studies [16], acting as a model organism for studying coronaviruses [17]. Although MHV belongs to betacoronavirus genus same with SARS-CoV-2, they are still taking different receptors; CEACAM1a for MHV and ACE2 for SARS-CoV-2 [16]. Nonetheless, MHV, which is subgenus Embecovirus (Lineage A), could offer metabolic insights into SARS-CoV-2 because MHV shares a common genus with SARS-CoV, SARS-CoV-2, and Middle East respiratory syndrome-related coronavirus (MERS-CoV). Indeed, MHV [18–20] and SARS-CoV-2 [21–23] induce similar clinical symptoms in hosts including brain, lung, and intestine. As a prototypical laboratory coronavirus, MHV has been studied in respiratory, enteric, neurological research for SARS-CoV-2 and conducted in low biosafety level [24]. Particularly, JHM strain is frequently studied for coronavirus pathogenicity and immunology [24]. Therefore, in this study, metabolic intervention was investigated as the therapeutic strategy against SARS-CoV-2 in MHV-infected DBT cells [25] in which glucose metabolism is highly vigoront due to organ characteristic of brain. By activating the TCA cycle with supplements including pyruvate and fatty acids, MHV replication was strongly suppressed. Furthermore, by increasing TCA coenzymes with nicotinamide riboside (NR) supplement, host defense was enforced against MHV.

## Results

### Alteration of host metabolism upon MHV infection

**Fig. 1:**
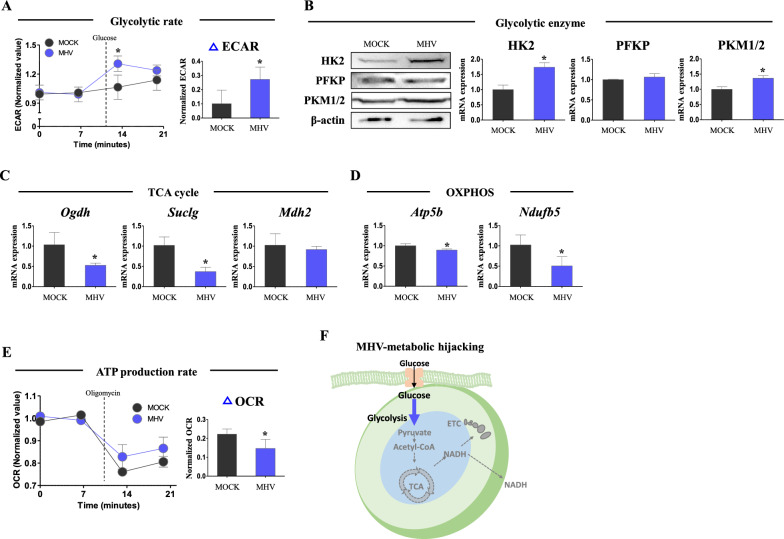
Host metabolic hijacking by MHV. Cells were infected by MHV (MOI 0.05) for 7 h. **A** Extracellular acidification rate measured by flux analyzer. Values were normalized by baseline value. Glucose (25 mM) was added for measurements. **B** Western blot analysis and quantification of glycolysis enzymes. β-actin was used for an internal control. **C** mRNA expression of TCA cycle enzymes. *Rplp0* was used for an internal control. **D** mRNA expression of ETC enzymes. *Rplp0* was used for an internal control. **E** Oxygen consumption rate measured by flux analyzer. Values were normalized by baseline value. Oligomycin (2 µM) was added for measurements. **F** Illustration of metabolic hijacking by MHV infection. Values represent means ± SD. *p < 0.05. Student’s t-test was performed. All experiments were repeated at least 3 times

Virus altered host metabolism, metabolic hijacking, has been investigated in diverse kinds of viruses, and particularly, glycolysis is shown to be increased by virus infection [26]. Furthermore, though the virus relies on host metabolism for its survival and the host produces most of its energy in mitochondria, mitochondrial disruption by various kinds of viruses has been observed [27]. To investigate the metabolic hijacking in MHV infection (MOI 0.05), we analyzed metabolic profile in early time point on 7 h post inoculation (hpi). In extracellular acidification rate (ECAR), glycolysis rate was 2.69-fold increased (*p *< 0.05), in MHV infected group compared to mock infected group (Fig. 1A). In Western blot, the levels of rate-limiting enzymes of glycolysis, HK2 and PKM1/2, were significantly increased (*p*<0.05, 1.74- and 1.36- fold vs. MOCK group) in MHV infected group (Fig. 1B). Conversely, mRNA expression of TCA cycle genes, *Ogdh* and *Suclg*, was down-regulated (*p *< 0.05, 50.9 and 36.3%, respectively, vs. MOCK group) by MHV infection (Fig. 1C). Furthermore, mRNA expression of oxidative phosphorylation (OXPHOS) genes, *Atp5b* and *Ndufb5*, was significantly decreased (*p *< 0.05, 89.3 and 49.7%, respectively, vs. MOCK group) by MHV infection (Fig. 1D). Consistently, ATP production rate was suppressed (*p *< 0.05, 66%, respectively, vs. MOCK group) in MHV group, suggesting host’s mitochondrial metabolism is impaired (Fig. 1E). In these results, it was observed that MHV infection increases glycolysis rate-limiting enzymes and ECAR, reflecting overall induction of glycolysis. On the contrary, MHV infection suppressed mitochondrial metabolism with low mitochondrial metabolic gene expression and reduced ATP production rate. MHV-metabolic hijacking is described in Fig. 1F.

### MHV requires glycolysis for its replication

**Fig. 2:**
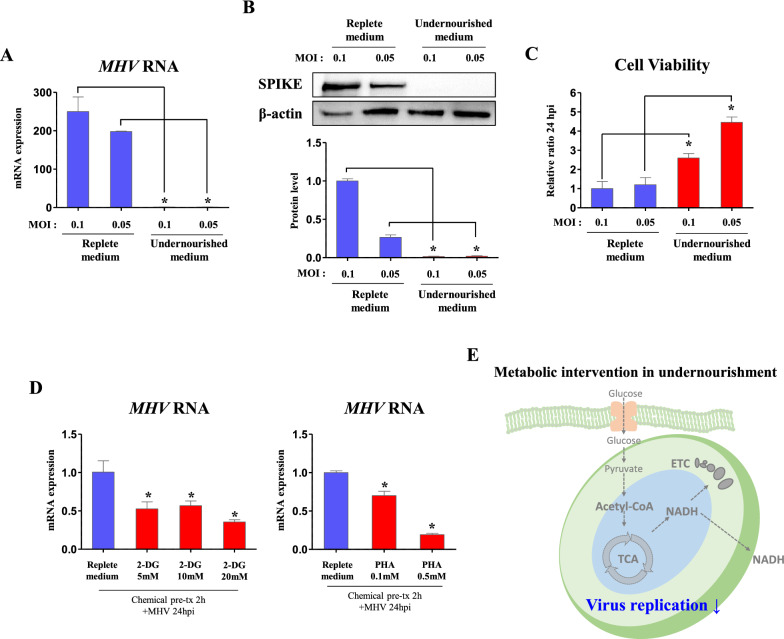
Inhibition of cellular glycolysis suppresses viral replication. Cells were infected by MHV (MOI 0.1 and 0.05) for 24 h. **A** RNA expression of *MHV* in cells incubated in replete (glucose 450 mg/dl, pyruvate 0.5 mM) or undernourished medium (glucose 50 mg/dl, pyruvate 0 mM). *Rplp0* was used for an internal control. **B** Western blot analysis and quantification of SPIKE in cells incubated in replete or undernourished medium. β-actin was used for an internal control. **C **Viability of cells incubated for 24 h with medium collected from MHV-infected cells which were treated as indicated. **D** RNA expression of *MHV* in cells (24 hpi, MOI 0.05) pre-treated with glycolysis inhibitors (2-Deoxyglucose, 2-DG; Phenylalanine, PHA, 2 h) in replete medium (glucose 450 mg/dl, pyruvate 0.5 mM). *Rplp0* was used for an internal control. **E** Illustration of metabolic intervention induced by depletion of cellular energy. Values represent means ± SD. *p < 0.05. One-way ANOVA followed by a tukey’s multiple comparison test. All experiments were repeated at least 3 times

Glucose is lysed by glycolysis and produces acetyl-CoA to run TCA cycle. Then, TCA cycle provides NADH to electron transport chain (ETC) for ATP production in mitochondria. As glycolysis and mitochondrial metabolism are sequential process following glucose usage [28], it was questioned how host will respond to virus when both of glycolysis and mitochondrial metabolism are almost turned off. To investigate, we infected DBT cells with MHV for 1 h and incubate them in replete medium (glucose 4500 mg/l, pyruvate 0.5 mM) or undernourished medium (glucose 500 mg/l, w/o pyruvate). When *MHV* copies were analyzed on 24 hpi, we could observe very low *MHV* RNA expression (> 1% of replete medium) in undernourished medium on both moi (*p *< 0.05, Fig. 2A). In Western blot, protein levels of SPIKE was strongly suppressed (*p *< 0.05, 1.45 and 6.77%, respectively) in MOI 0.1 and MOI 0.05 MHV-infected cells of undernourished medium compared to those of replete medium (Fig. 2B). Then, cell culture medium was treated to fresh DBT cells, and viability of cells was measured after 24 h. As a result, cell viability was significantly increased (*p *< 0.05, 2.6- and 3.71-fold) in medium of undernourished MOI 0.1 and MOI 0.05 MHV-infected cells compared to those of replete cells (Fig. 2C). Through these results, it was confirmed that MHV replication and translation are strongly suppressed in undernourished medium, suggesting that basal cellular nutrition is needed for viral replication. As an undernourished medium contains very low glucose compared to replete medium, cells in the undernourished medium might primarily lack glycolysis and glucose-derived metabolism. Indeed, glycolysis inhibition reduced glycolytic-dependent oxidative phosphorylation and ATP production rates (Additional file 1: Fig. S1), which may be due to reduced glucose-derived ETC supply. Therefore, it can be assumed that inhibition of glycolysis or ETC involves in viral suppression.

To investigate whether glycolysis affects viral replication, we firstly treated glycolysis inhibitors, 2-deoxyglucose (2-DG) [29] and phenylalanine (PHA) [30]. For experiment, DBT cells were pre-incubated with medium containing glycolysis inhibitors for 2 h and infected with MHV (MOI 0.05). RNA copies of *MHV* were significantly decreased (*p* < 0.05, 52.4, 56.6, and 35.4%, respectively) in 5, 10, and 20 mM of 2-DG treated cells compared to those of vehicle treated cells (Fig. 2D). Likewise, *MHV* RNA expression was significantly decreased (*p*<0.05, 70 and 19.2%, respectively) in 0.1 and 0.5 mM of PHA treatments (Fig. 2D). Accordingly, it was observed that glycolysis inhibition restrains virus replication, which is consistent to previous SARS-CoV-2 observation [31]. The metabolic intervention by undernourishment in MHV-infected cells is described in Fig. 2E.

#### ETC inhibition suppresses MHV replication

**Fig. 3:**
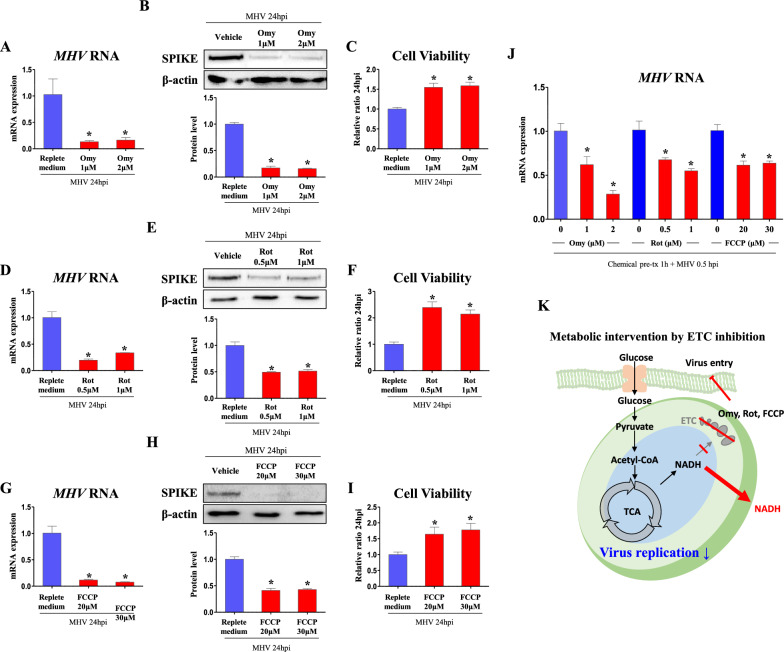
ETC inhibition stongly suppresses viral replication. Cells were infected by MHV (MOI 0.05) for 24 h. Chemicals were treated after serum free medium is changed to FBS containing medium. **A**, **D**, **G** RNA expression of *MHV* in cells treated with ETC inhibitors (Oligomycin, Omy; Rotenone, Rot; FCCP) in replete medium (glucose 450 mg/dl, pyruvate 0.5 mM). *Rplp0* was used for an internal control. **B**, **E**, **H** Western blot analysis and quantification of SPIKE in cells treated with ETC inhibitors (Omy, Rot, FCCP) in replete medium (glucose 450 mg/dl, pyruvate 0.5 mM). β-actin was used for an internal control. **C**, **F**, **I** Viability of cells incubated for 24 h with medium collected from MHV-infected cells which were treated as indicated. **J** RNA expression of *MHV* in cells pre-treated with ETC inhibitors (indicated doses) 1 h before MHV infection. Cells were washed and harvested in 0.5 hpi. *Rplp0* was used for an internal control. **K** Illustration of metabolic intervention induced by ETC inhibition. Values represent means ± SD. *p < 0.05. One-way ANOVA followed by a tukey’s multiple comparison test was performed. All experiments were repeated at least 3 times

Next, ETC was inhibited with multiple inhibitors for each complex. Firstly, to inhibit the last step of ETC, oligomycin, ATP synthase inhibitor, was treated to cell. As a result, 1 and 2 µM of oligomycin treated cells decreased (*p *< 0.05, 13 and 16%, respectively) RNA copies of *MHV* compared to vehicle treated cells (Fig. 3A). In Western blot, protein levels of SPIKE were suppressed (*p *< 0.05, 17.2 and 15.8%, respectively, vs. vehicle treated cells) in 1 and 2 µM of oligomycin treated cells (Fig. 3B). In line with virus protein levels, when virus infected cell culture medium was treated to fresh DBT cells, cell viability was increased (*p *< 0.05, 1.55- and 1.59-fold, respectively, vs. vehicle treated cells) in medium of 1 and 2 µM of oligomycin treated cells (Fig. 3C).

Furthermore, to inhibit the first step of ETC, rotenone, complex 1 inhibitor, was treated to cell. As a result, 1 and 2 µM of rotenone treated cells decreased (*p *< 0.05, 13 and 16%, respectively) RNA copies of *MHV* compared to vehicle treated cells (Fig. 3D). In Western blot, protein levels of SPIKE were suppressed (*p *< 0.05, 17.2 and 15.8%, respectively, vs. vehicle treated cells) in 1 and 2 µM of rotenone treated cells (Fig. 3E). When virus infected cell culture medium was treated to fresh DBT cells, cell viability was increased (*p *< 0.05, 1.55- and 1.59-fold, respectively, vs. vehicle treated cells) in medium of 1 and 2 µM of rotenone treated cells (Fig. 3F).

For universal inhibition of ETC, proton gradient was flattened by ionophore, FCCP. As a result, 20 and 30 µM of FCCP treated cells decreased (*p *< 0.05, 11.7 and 7.62%, respectively) RNA copies of *MHV* compared to vehicle treated cells (Fig. 3G). In Western blot, protein levels of SPIKE were suppressed (*p *< 0.05, 41.2 and 42.8%, respectively, vs. vehicle treated cells) in 20 and 30 µM of FCCP treated cells (Fig. 3H). When virus infected cell culture medium was treated to fresh DBT cells, cell viability was increased (*p *< 0.05, 1.64- and 1.78-fold, respectively, vs. vehicle treated cells) in medium of 20 and 30 µM of FCCP treated cells (Fig. 3I). Meanwhile, the viral suppression by ETC inhibitors is not solely affected by its decreased replication, because pre-treatment of ETC inhibitors in cells significantly reduced intracellular *MHV* RNA expression on just 0.5 hpi (Fig. 3J), implying intracellular virus entry was already decreased before virus replication. Illustration of metabolic intervention by ETC inhibition in MHV-infected cells is depicted in Fig. 3K.

### TCA cycle activation by pyruvate and fatty acids suppresses replication of MHV

In biochemistry, glucose is well known to be lysed to pyruvate via glycolysis, and pyruvate is oxidized to acetyl-CoA. Acetyl-CoA enters and runs the TCA cycle through a series of steps, producing NADH and FADH_2_ which act as ingredients for ATP production in ETC. As ETC inhibition suppressed viral replication, it was further investigated whether the metabolic intervention before ETC will follow the tendency of ETC regulation. Herein, we tested whether TCA cycle regulation will affect viral replication.

**Fig. 4:**
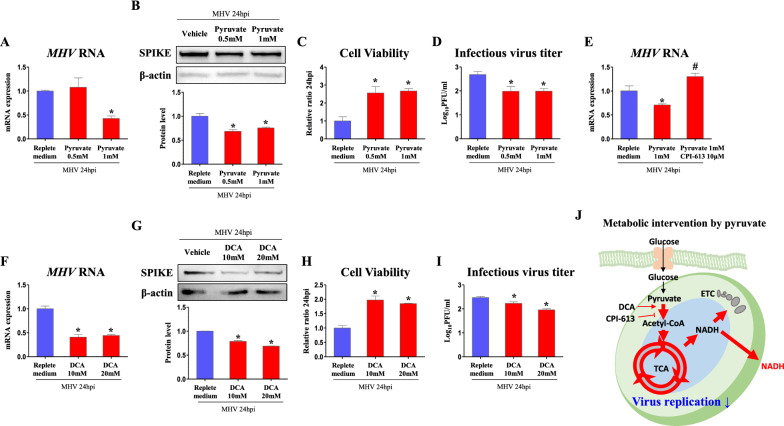
Pyruvate oxidation decreases viral replication via induction of TCA cycle. Cells were infected by MHV (MOI 0.05) for 24 h. Metabolite and chemicals were treated after serum free medium is changed to FBS containing medium. **A**, **E**, **F** RNA expression of *MHV* in cells treated with pyruvate, pyruvate oxidation inhibitor (CPI-613), and pyruvate oxidation activator (Dichloroacetate, DCA) in replete medium (glucose 450 mg/dl, pyruvate 0.5 mM). *Rplp0* was used for an internal control. **B**, **G** Western blot analysis and quantification of SPIKE in cells treated with pyruvate and pyruvate oxidation activator in replete medium (glucose 450 mg/dl, pyruvate 0.5 mM). β-actin was used for an internal control. **C**, **H** Viability of cells incubated for 24 h with medium collected from MHV-infected cells which were treated as indicated. **D**, **I** Infectious virus titer (Log_10_PFU/ml) measured by plaque forming assay. **J** Illustration of metabolic intervention induced by pyruvate and pyruvate oxidation. Values represent means ± SD. *p < 0.05 vs. vehicle. #*p *< 0.05 vs. pyruvate. One-way ANOVA followed by a tukey’s multiple comparison test. All experiments were repeated at least 3 times

Firstly, as glycolytic product, pyruvate was treated as precursor supplement for TCA cycle activation. As a result, RNA copies of *MHV* was suppressed (*p *< 0.05, 42.8% vs. non-supplementation) in 1 mM of pyruvate supplementation (Fig. 4A). Furthermore, protein levels of SPIKE were decreased (*p *< 0.05, 68.4 and 75.6%, respectively, vs. non-supplementation) following 0.5 and 1 mM of pyruvate supplementation (Fig. 4B). When cell viability was measured as aforementioned experiments, cells incubated with 0.5 and 1 mM of pyruvate supplemented medium survived in larger numbers (*p *< 0.05, 2.56- and 2.67-fold, respectively) compared to non-supplementation (Fig. 4C). Consistently, when plaque-forming unit (PFU) was measured in plaque-forming assay (PFA), 0.5 and 1 mM of pyruvate supplementaion reduced (*p *< 0.05, 74.1 and 74.2%, respectively) infectious virus titer (Fig. 4D). To demonstrate whether pyruvate is on-target TCA inducer for viral suppression, pyruvate supply was hindered by mitochondrial pyruvate dehydrogenase inhibitor, CPI-613. When pyruvate supply still decreased (*p *< 0.05, 70.5% vs. vehicle) RNA copies of *MHV*, CPI-613 and pyruvate co-treatment recovered *MHV* RNA expression (*p *< 0.05, 1.84-fold vs. pyruvate treatment) to those of vehicle treated cells (Fig. 4E). Given that pyruvate oxidation limits viral replication, we activated conversion from pyruvate to acetyl-CoA by treating dichloroacetate (DCA), pyruvate dehydrogenase kinase inhibitor. As a result, RNA copies of *MHV* were significantly suppressed (*p *< 0.05, 40.8 and 44.3%, respectively) in 10 and 20 mM of DCA treated cells compared to those of vehicle treated cells (Fig. 4F). In Western blot, protein levels of SPIKE were decreased (*p *< 0.05, 78.8 and 68.8%, respectively) in 10 and 20 mM of DCA treated cells (Fig. 4G). Accordingly, cell viability was increased (*p *< 0.05, 1.97- and 1.85-fold, respectively) when medium of 10 and 20 mM of DCA treated cells was inoculated (Fig. 4H). When PFU was measured, 10 and 20 mM of DCA treatments reduced (*p *< 0.05, 90.2 and 79.4%, respectively) infectious virus titer (Fig. 4I). Metabolic intervention by pyruvate oxidation in these experiments is illustrated in Fig. 4J, showing that activation of TCA cycle by pyruvate supply suppresses MHV replication.

**Fig. 5:**
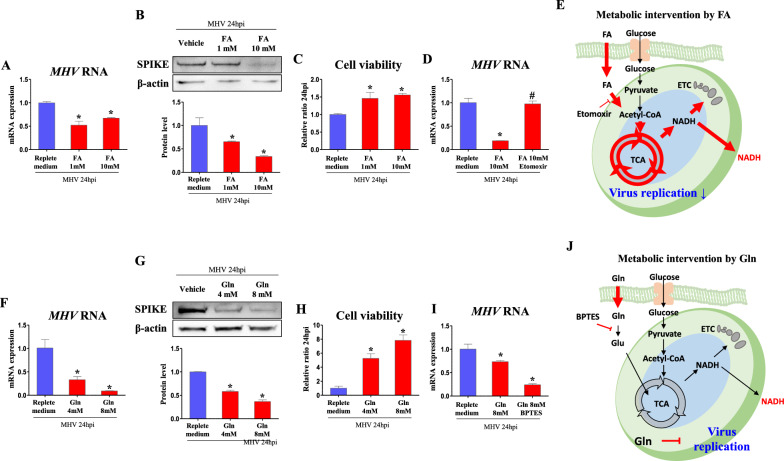
Fatty acids suppresses viral replication via induction of TCA cycle. Cells were infected by MHV (MOI 0.05) for 24 h. Metabolites and chemicals were treated after serum free medium is changed to FBS containing medium. **A**, **D**, **F**, **I** RNA expression of *MHV* in cells treated with fatty acid (palmitic acid 330 µM and oleic acid 660 µM for fatty acid 1 mM), fatty acid oxidation inhibitor (etomoxir), glutamine, and glutaminase inhibitor (BPTES) in replete medium (glucose 450 mg/dl, pyruvate 0.5 mM). *Rplp0* was used for an internal control. **B**, **G** Western blot analysis and quantification of SPIKE in cells treated with fatty acid and glutamine in replete medium (glucose 450 mg/dl, pyruvate 0.5 mM). β-actin was used for an internal control. **C**, **H** Viability of cells incubated for 24 h with medium collected from MHV-infected cells which were treated as indicated. **E**, **J** Illustration of metabolic intervention induced by fatty acid and glutamine. Values represent means ± SD. *p < 0.05 vs. vehicle. #*p *< 0.05 vs. FA. One-way ANOVA followed by a tukey’s multiple comparison test. All experiments were repeated at least 3 times

Following these results, other endogenous metabolites were tested whether they decrease MHV replication by activation of TCA cycle. Except for glucose, fatty acid was possible candidate for activating TCA cycle as they are oxidized to acetyl-CoA through fatty acid oxidation. When we treated fatty acids (palmitic acid 330 µM and oleic acid 660 µM per fatty acids 1 mM), RNA copies of *MHV* were suppressed (*p *< 0.05, 52.1 and 67.2%, respectively, vs. non-supplementation) in 1 and 10 mM of fatty acids supplementation, suggesting viral replication was suppressed (Fig. 5A). In Western blot, protein levels of SPIKE were suppressed (*p *< 0.05, 65.3 and 33.8%, respectively, vs. non-supplementation) in 1 and 10 mM of fatty acids supplementation (Fig. 5B). In line with virus protein levels, cell viability was increased (*p *< 0.05, 1.46- and 1.56-fold, respectively, vs. non-supplementation) when medium of 1 and 10 mM of fatty acids supplementation was incubated (Fig. 5C). To further demonstrate whether fatty acid is on-target TCA inducer for viral suppression, etomoxir was treated as fatty acid oxidation inhibitor. Consistent to dose-dependent results, fatty acids supplementation substantially restrained (*p *< 0.05, 18.7%) viral replication compared to non-supplementation. Importantly, 10 µM of etomoxir treatment in fatty acids supplementation recovered expression of *MHV* RNA (*p *< 0.05, 5.2-fold vs. fatty acids supplementation) to those of non-supplementation (Fig. 5D). Accordingly, fatty acids might be on-target TCA cycle inducer suppressing viral replication, regarding etomoxir inhibits acetyl-CoA production from fatty acids (Fig. 5E).

In addition, another metabolite, glutamine, involves in TCA cycle as it converts alpha-ketoglutarate (α-KG). When glutamine was supplemented to medium, RNA copies of *MHV* were suppressed (*p *< 0.05, 32.9 and 9.14%, respectively) in 4 and 8 mM of glutamine supplementation compared to non-supplementation (Fig. 5F). In Western blot, protein levels of SPIKE were suppressed (*p *< 0.05, 58.2 and 36.8%, respectively, vs. non-supplementation) in 4 and 8 mM of glutamine supplementation (Fig. 5G). In line with virus protein levels, when virus infected cell culture medium was treated to fresh DBT cells, cell viability was increased (*p *< 0.05, 5.25- and 7.83-fold, respectively, vs. non-supplementation) in medium of 4 and 8 mM of glutamine supplementation (Fig. 5H). Glutaminase inhibitor, BPTES, was treated to confirm whether glutamine acts as an on-target TCA inducer to decrease viral replication. Unexpectedly, BPTES did not recover viral copies and further decreased (*p*<0.05, 32.8%) *MHV* RNA compared to glutamine treated cells (Fig. 5I). Therefore, it can be concluded that glutamine can suppress viral replication, but it is not mediated by TCA induction. As BPTES might increase intact glutamine by decreasing glutamine degradation to glutamate (Glu), it should be considered that high glutamine concentration inhibits viral replication regardless of TCA cycle (Fig. 5J).

### NR suppresses replication of MHV

**Fig. 6:**
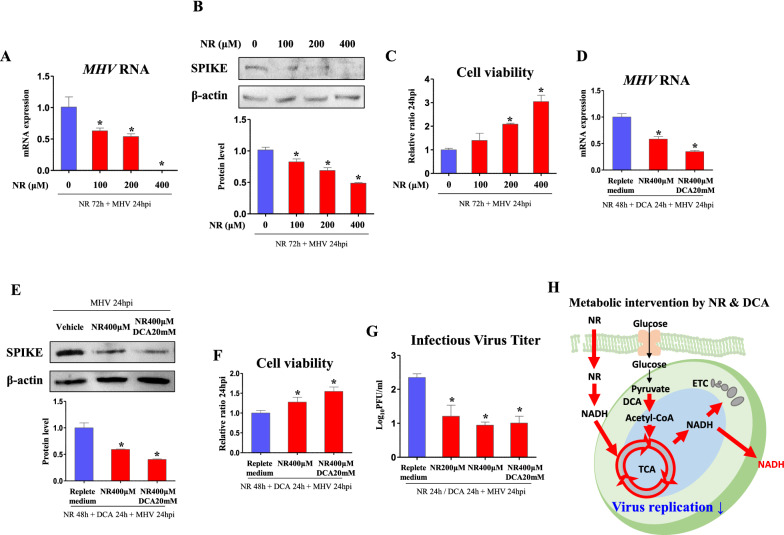
NR suppresses viral replication. Cells were infected by MHV (MOI 0.05) for 24 h following NR pre-treatment for indicated hours. Metabolite and chemicals were treated after serum free medium is changed to FBS containing medium. **A**, **D** RNA expression of *MHV* in cells treated with NR (indicated dose and time) and DCA (20 mM, 24 h) in replete medium (glucose 450 mg/dl, pyruvate 0.5 mM). *Rplp0* was used for an internal control. **B**, **E **Western blot analysis and quantification of SPIKE in cells treated with NR (indicated dose and time) and DCA (20 mM, 24 h) in replete medium (glucose 450 mg/dl, pyruvate 0.5 mM). β-actin was used for an internal control. **C**, **F** Viability of cells incubated for 24 h with medium collected from MHV-infected cells which were treated as indicated. **G** Infectious virus titer (Log_10_PFU/ml) measured by plaque forming assay. **H** Illustration of metabolic intervention induced by NR and DCA. Values represent means ± SD. *p < 0.05. One-way ANOVA followed by a tukey’s multiple comparison test. All experiments were repeated at least 3 times

Although those regulators for TCA cycle and ETC show anti-MHV effects, they were treated in high dose which may trigger toxicity for host. Therefore, we introduced NR, which serves as a TCA coenzyme supplier [32], as safe therapeutic agent for coronavirus as it has been tested in previous human clinical trial [33]. As a result, RNA copies of *MHV* were significantly suppressed (*p *< 0.05, 62.5, 53.4, and 0.04%, respectively) in 100, 200, and 400 µM of NR treated cells compared to those of vehicle treated cells (Fig. 6A). In Western blot, protein levels of SPIKE (*p *< 0.05, 81.4, 67.8, and 48.1%, respectively) was decreased in 100, 200, and 400 µM of NR treated cells (Fig. 6B). Accordingly, cell viability was increased (*p *< 0.05, 2.09- and 3.04-fold, respectively) when medium of 200 and 400 µM of NR treated cells was inoculated (Fig. 6C). To improve the effect of NR, DCA was co-treated to stimulate TCA cycle. As a result, RNA copies of *MHV* was significantly suppressed (*p *< 0.05, 58.2%) in 400 µM of NR treated cells and further decreased (*p *< 0.05, 34.6%) in DCA-NR co-treated cells (Fig. 6D). In Western blot, protein levels of SPIKE was significantly suppressed (*p *< 0.05, 59.3%) in 400 µM of NR treated cells and further decreased (*p *< 0.05, 40.4%) in DCA-NR co-treated cells (Fig. 6E). Cell viability was increased (*p *< 0.05, 1.28- and 1.55-fold, respectively) when medium of NR and DCA-NR treated cells was inoculated, compared to those of vehicle treated cells (Fig. 6F). However, DCA-NR did not result in improvement in cell viability compared to NR. When PFA was analyzed, infectious virus titer was decreased (*p *< 0.05, 51.4, 40.3, and 42.8%, respectively) in 200 and 400 µM of NR treatments, and NR-DCA co-treatments, compared to those of vehicle treated cells (Fig. 6G). However, DCA-NR could not decrease PFU compared to NR, because they may have a little synergistic effect that only can affect total viral replication. Metabolic intervention triggered by NR and DCA in MHV-infected cells is illustrated in Fig. 6H. These results offer novel therapeutic potential of NR against coronavirus infection which both presents viral suppressive effect and low host-toxicity.

## Discussion

MHV is betacoronaviridae which has been used for coronavirus study very frequently as it possesses similarity with SARS-CoV-2 in various aspects. In a previous study, it has been shown that MHV shares inhibitory effects for virus entry with SARS-CoV-2 [34]. Furthermore, MHV is closely related to pro-inflammatory response, similar to SARS-CoV-2, including induction of cytokines such as TNF and IL-1β, and IFN-gamma [35]. Additionally, MHV and SARS-CoV-2 have typical decay patterns in an environment [36]. Studying common characteristics between SARS-CoV-2 and MHV is important for developing SARS-CoV-2 therapy in the clinic. For example, previous study elucidated the importance of antibody production during MHV infection [37], which led to further therapeutic investigation using monoclonal antibodies against viral spike, matrix, and nucleocapsid proteins for viral clearance [38]. As those approaches were valid in SARS-CoV-2 study [39], it should be noted that basic research for MHV could suggest a possible therapeutic approach for SARS-CoV-2.

Previous pieces of literature observed that induction of glycolysis [12] and suppression of OXPHOS [40] in immune cells lead to pro-inflammatory cytokine storm with a decrease of host-defensive cytokine, interferon-gamma (IFN-γ). It was also shown that the induced glycolysis in PBMC of COVID-19 patients is a compensatory response from mitochondrial respiration deficit [41], suggesting mitochondrial regulation might be important for viral resistance. While the metabolic alteration in SARS-CoV-2 infected immune cells has been well studied, the metabolism of infected non-immune cells was not investigated though the host immune system acts following viral replication in epithelial cells [42]. Hence, our study investigated the physiology of coronavirus on non-immune cells, mainly focusing on the regulation of viral replication by exerting metabolic intervention on TCA cycle. As a result of metabolic hijacking by MHV, glycolysis was increased while TCA cycle and OXPHOS were decreased. Consistent to immune cells and lung cells [12, 31], we found that replication of MHV in brain cell can be suppressed by glycolysis inhibition. Furthermore, chemical inhibition of ETC strongly suppressed MHV replication though the inhibitors cannot be applied in the clinic due to toxicity as also shown in Additional file 1: Fig. S2. Contrary to glycolysis and ETC, metabolic and chemical activation of the TCA cycle by pyruvate, fatty acids, and NR suppressed the replication of MHV. When glucose-derived pyruvate can serve as main substrate for lung TCA cycle [43], we observed that non-glucose metabolites also could induce the TCA cycle of human bronchial epithelial Calu-3 cells (Additional file 1: Fig. S3), suggesting possibility for their use for SARS-CoV-2 amelioration in lung. Particularly, NR may present high potential as a clinical therapeutic agent against coronavirus replication with its known safety in humans.

In fundamental nutritional deprivation triggered with an undernourished medium, which contains very low glucose (50 mg/dl) without pyruvate, cells can exploit little glycolysis. Hence, undernourishment and glycolysis inhibition similarly inhibited viral replication. Undernourishment also reduces glucose-derived subtrates for ETC complexes which produce cellular energy by oxidative phosphorylation [44]. Therefore, when cells were treated with ETC complex inhibitors and ion-gradient destroyer, they were remarkably resistant to viral infection similar to undernourished cells. These results could be assumed that cells become resistant to viral infection when cellular glycolysis and energy production are inhibited. However, these chemicals are yet to be applied in clinic since the data is lacking whether host can be protected from toxicity of those drugs.

Cellular energy production depends on mitochondria, and glucose, long-chain fatty acids (LCFA), and glutamine are main sources for mitochondrial metabolism, including TCA cycle. As a major nutrition source, glucose is oxidized to pyruvate by glycolysis, and pyruvate is oxidized to acetyl-CoA to run TCA cycle. Although inhibition of glycolysis, which can produce pyruvate, suppressed MHV replication, pyruvate and pyruvate oxidation suppressed MHV replication. Additionally, as viral replication was increased by inhibition of pyruvate oxidation, the viral-inhibitory effect of pyruvate supply could be involved with acetyl-CoA production and its mediation in the TCA cycle. In a study, it has been well shown that SARS-CoV-2 replication was increased by glucose, while not increased by pyruvate [12], suggesting SARS-CoV-2 virulence is increased only by glycolytic steps before pyruvate production. Similarly, our results show that MHV replication can be induced by glucose metabolic steps before pyruvate production, whereas it is suppressed by pyruvate and pyruvate oxidation step.

We further evaluated whether LCFA (palmitic acid and oleic acid) and glutamine, TCA cycle substrates, derived mitochondrial metabolisms affect virulence. While LCFA and glutamine both participate in TCA cycle, the metabolic pathway is different; LCFA are oxidized into acetyl-CoA by fatty acid oxidation [45], whereas glutamine is converted to alpha-ketoglutarate [46]. In our results, treatments of LCFA and glutamine suppressed viral replication. Etomoxir treatment, which inhibits LCFA conversion to acetyl-CoA, also showed recovery of viral replication from LCFA treatment, suggesting TCA cycle induction is necessary for their metabolic intervention. Nonetheless, an inhibitor of glutaminase could not show consistency in subsequent results, suggesting glutamine is not an on-target metabolite for TCA cycle induction for viral suppression.

Although TCA cycle products are linked to activation of ETC, TCA cycle and ETC are metabolically different as NADH is either produced or consumed by those respective processes. Additionally, ATP production by ETC lowers NADH level, whereas ETC inhibition heightens NADH level. Hence, MHV suppression by TCA cycle activation and ETC inhibition must be related to increase of NADH level. According to previous literature, NAD^+^ level was suggested to be inversely correlated to SARS-CoV-2 induced death as elderly people decreases NAD^+^ [47]. Mechanistically, in silico analysis of the study predicted molecular docking of NAD^+^ and NADH with the main protease of SARS-CoV-2 [47], which is responsible for the control of viral replication and life cycle [48]. Furthermore, one hypothesis suggested that NAD^+^ depletion leads to SirT1 inactivation leading to a hyperinflammatory state of the host [49] whereas another theoretical study also suggested that NAD+ inhibits PARP-1 and thereby prevents pro-inflammatory cytokine [50]. Based on previous literatures, our study used NR to increase NAD^+^ or NADH level, because NR is known to synthesize NAD^+^ [51], and observed the significant suppression of viral replication by NR. Since NAD^+^ can be converted to NADH by the TCA cycle, DCA and NR synergistically activated the TCA cycle and stimulated the suppression of viral replication. Although metabolites can lower the cellular pH, we could not observe a significant relevance between pH of metabolites-incubated cell culture medium and viral suppression (Additional file 1: Fig. S4), suggesting NR suppresses viral replication regardless of pH fluctuation in the host cell. We suggest NR, the supplier of TCA coenzyme and activator of TCA cycle, as a high potential anti-coronavirus drug.

In clinical therapy, oral supplementation of L-glutamine reduced hospitalized time of COVID-19 patients [52], and a nasal spray of sodium pyruvate suppressed symptoms of COVID-19 in the respiratory system (NCT04824365). Our findings provide basic evidence supporting those clinical reports. Furthermore, we highlight the therapeutic potential for activating TCA cycle to reduce hospitalized patients’ severity, since previous studies observed TCA cycle impairment in SARS-CoV-2 infection [14, 15]. Particulary, as the safe metabolite, we suggest NR as a novel therapeutic strategy for COVID-19 that can immediately be used in the clinic.

## Conclusions

In present study, we showed that TCA cycle activation by chemicals and metabolites restrains replication of MHV. Through these results, we suggest novel therapeutic approach by exerting metabolic intervention to TCA cycle, particularly with use of NR, against SARS-CoV-2.

## Methods

### Reagents

Reagents used for experiments: 2-deoxy-d-glucose (D0051, TCI), l-phenylalanine (P0134, TCI), oligomycin A (75351, Sigma-Aldrich), rotenone (R8875, Sigma-Aldrich), FCCP (C2920, Sigma-Aldrich), sodium pyruvate (11360070, Gibco), sodium dichloroacetate (347795, Sigma-Aldrich), palmitic acid (P0500, Sigma-Aldrich), oleic acid (O1008, Sigma-Aldrich), l-glutamine (LS002-01, Welgene), CPI-613 (SML0404, Sigma-Aldrich), (+)-Etomoxir sodium salt hydrate (E1905, Sigma-Aldrich), BPTES (SML0601, Sigma-Aldrich), nicotinamide riboside chloride (SMB00907, Sigma-Aldrich),

### RNA isolation, reverse transcription, and qRT-PCR

RNA was extracted from DBT cells. After homogenization with the Trizol reagent, homogenates were mixed with chloroform and centrifuged to collect supernatant. Following incubation of supernatant with isopropanol, RNA pellets were washed with ethanol and dissolved in DEPC-treated water. cDNA was synthesized with 1 µg of total RNA by using a reverse transcriptase kit (SG-cDNAS100, Smartgene, Daejeon, Korea) according to the manufacturer’s protocol. Quantitative PCR (real-time PCR) was carried out using specific primers (Table 1), Excel Taq Q-PCR Master Mix (SG-SYBR-500, Smartgene), and Stratagene Mx3000P (Agilent Technologies) equipped with a 96-well optical reaction plate. All experiments were repeated in triplicates, and the mRNA values were calculated based on the cycle threshold and monitored for a melting curve.

**Table 1:** Primers used for real-time PCR

Gene name	Upper primer (5′–3′)	Lower primer (5′–3′)	Species
*Ogdh*	AAT GCT GAG CTG GCC TGG TG	TCA GGT GTG TTT TCT TGT TGC C	Mouse
*Suclg2*	CTG TGC CAT CAT TGC CAA CG	ATG GGG AGT CCG CTG CTC TT	Mouse
*Mdh2*	ATG CTG GAG CCC GCT TTG TC	CAG GGA TAG CCT CGG CAA TC	Mouse
*Atp5b*	GTA CTG GAT TCA GGG GCA CC	CTA TGA ACT CAG GAG CCT CAG C	Mouse
*Ndufb5*	CGA GCT TGC AGA AAT CCC AGA AGG C	GTC CAT CAC CTC GGG CAC GCA TCA G	Mouse
*Rplp0*	GCA GCA GAT CCG CAT GTC GCT CCG	GAG CTG GCA CAG TGA CCT CAC ACG G	Mouse
*MHV*	CAC GAG CCG TAG CAT GTT TA	GAA GCT CCA CCA GCT ACC AG	Mouse
*Rplp0*	TCG ACA ATG GCA GCA TCT AC	GCC TTG ACC TTT TCA GCA AG	Human
*Ogdh*	GGA ATC AGC ACT TCC TCT GC	AGG GTC CGT TTC TCC TCA TT	Human
*Idh3a*	AAC ATC ATG CGG ATG TCA GA	CAA TGT TGC CAC TTG GTG TC	Human

### Western blot

Protein was extracted from DBT cells by homogenization with T-PER buffer. After electrophoresis, gels were blotted to a PVDF membrane, and the membrane was blocked and incubated with primary antibodies. After overnight incubation, the membranes were washed and incubated with secondary antibodies (Goat anti-Rabbit IgG HRP; Catalog # 31460, Goat anti-Mouse IgG HRP; Catalog # 31430, Thermo Fisher Scientific). The bands were observed with ECL solution (XLS025-0000, Cyanagen) after washing three times.

The following primary antibodies were used: Glycolysis Antibody Sampler Kit (CST, #8337), β-actin (sc-130,656, Santa Cruz Biotechnology) and SPIKE (mouse monoclonal, homemade).

### Cell culture

All cell culture reagents were purchased from Welgene (Gyungsan, Korea). DBT mouse brain tumor cells were maintained at 37 °C in a 5% CO_2_ atmosphere in DMEM (Welgene, LM001-05) supplemented with 5% (vol/vol) fetal bovine serum, penicillin (100 U/mol), and streptomycin (100 µg/ml). For virus inoculation, serum-free DMEM was incubated with virus for 1 h and medium was changed to DMEM containing FBS. pH was measured by using BCECF, AM (2’,7’-Bis-(2-Carboxyethyl)-5-(and-6)-Carboxyfluorescein, Acetoxymethyl Ester) (B1170, Thermo Fisher Scientific).

### Cell viability

Cell viability was measured by staining cells with trypan blue. Viable cells were counted and calculated relative to control group.

### Measurements of ECAR and OCR

MHV-infected DBT cells were cultured in seahorse cell culture plate. Cells were then starved with DMEM medium (glucose 4500 mg/l, w/o FBS) for 5 h to remove endogenous hormones and incubated with low glucose-DMEM medium (glucose 500 mg/l, w/o FBS) for 1 h. Cells were decarboxylated for 40 min to 1 h in XFp medium (103575-100, Agilent technologies) containing same amount of glutamine, sodium pyruvate, and glucose compared to medium in which cells were grown. For glycolysis measurement, glucose (25 mM) was used and ECAR was measured. For ATP production measurement, oligomycin (2 µM) was used and OCR was measured. Seahorse XFp analyzer (Agilent technologies) and Seahorse XFp, XFp FluxPak (103022-100, Agilent technologies) were used for experiment.

### Statistical analysis

Data are reported as mean ± standard deviation. Differences between the means were assessed using Student’s *t*-test and one-way ANOVA followed by a tukey’s multiple comparison test. All statitiscal analyses were performed using the GraphPad Software (GraphPad Inc., San Diego, CA, USA).

## Supplementary Information

**Additional file 1: Figure S1.** Suppressed mitochondrial energy metabolism by glycolysis inhibitors. Extracellular acidification rates and oxygen consumption rates were measured before and after oligomycin treatment. Rate changes were normalized relative to baseline. DBT cells were treated for 30 h as indicated; 2-DG (2-deoxyglucose, 1mM), PHA (phenylalanine, 0.5 mM). Values represent means ± SD. *, *p* < 0.05. One-way ANOVA followed by a tukey's multiple comparison test was performed. All experiments were repeated at least 3 times. **Figure S2.** Cell viability of DBT cells treated for 30 h as indicated; 2-DG (2-deoxyglucose, 1 mM), PHA (phenylalanine, 0.5 mM), Pyr (pyruvate, 0.5 mM), DCA (dichloroacetate, 20 mM), NR (nicotinamide riboside, 400 μM), Omy (oligomycin, 2 μM), Rot (rotenone, 1 μM), FCCP (30 μM), FA (fatty acids, 10 mM), Gln (glutamine, 8 mM). Values represent means ± SD. *, *p* < 0.05. One-way ANOVA followed by a tukey’s multiple comparison test was performed. All experiments were repeated at least 3 times. **Figure S3.** Gene expression of TCA cycle in Calu-3 cells treated for 24 h as indicated; FA (fatty acids, 10 mM), NR (200 μM). Rplp0 was used for an internal control. Values represent means ± SD. *, *p* < 0.05. Student’s *t*-test was performed. All experiments were repeated at least 3 times. **Figure S4.** Intracellular pH of DBT cells treated for 24 h as indicated; Replete (Rep; glucose 450 mg/dl, pyruvate 0.5 mM), undernourished (glucose 50 mg/dl, pyruvate 0 mM), 2-DG (2-deoxyglucose, 1 mM), PHA (phenylalanine, 0.5 mM), Pyr (pyruvate, 0.5 mM), DCA (dichloroacetate, 20 mM), Omy (oligomycin, 2 μM), Rot (rotenone, 1 μM), FCCP (30 μM), FA (fatty acids, 10 mM), Gln (glutamine, 8 mM). Values represent means ± SD. *, *p* < 0.05. Student’s *t*-test and one-way ANOVA followed by a tukey’s multiple comparison test were performed. All experiments were repeated at least 3 times.

## Data Availability

The datasets used and/or analyzed during the current study are available from the corresponding author on reasonable request.

## Abbreviations

MHV: Mouse hepatitis virus
HK2: Hexokinase 2
PFKP: Phosphofructokinase, platelet
PKM1/2: Pyruvate kinase M1/M2
Ogdh: Alpha-ketoglutarate dehydrogenase
Suclg: Succinyl-CoA ligase
Mdh2: Malate dehydrogenase 2
Atp5b: ATP synthase F1 subunit beta, mitochondrial
Ndufb5: NADH:Ubiquinone Oxidoreductase Subunit B5
OCR: Oxygen consumption rate
ECAR: Extracellular acidification rate

## References

[1] Hu B, Guo H, Zhou P, Shi ZL (2021). Characteristics of SARS-CoV-2 and COVID-19. Nat Rev Microbiol.

[2] V’Kovski P, Kratzel A, Steiner S, Stalder H, Thiel V (2021). Coronavirus biology and replication: implications for SARS-CoV-2. Nat Rev Microbiol.

[3] Beigel JH, Tomashek KM, Dodd LE, Mehta AK, Zingman BS, Kalil AC, Hohmann E, Chu HY, Luetkemeyer A, Kline S (2020). Remdesivir for the treatment of covid-19 - final report. N Engl J Med.

[4] Spinner CD, Gottlieb RL, Criner GJ, Arribas Lopez JR, Cattelan AM, Soriano Viladomiu A, Ogbuagu O, Malhotra P, Mullane KM, Castagna A (2020). Effect of remdesivir vs standard care on clinical status at 11 days in patients with moderate COVID-19: a randomized clinical trial. JAMA.

[5] Group RC (2020). Lopinavir-ritonavir in patients admitted to hospital with COVID-19 (RECOVERY): a randomised, controlled, open-label, platform trial. Lancet.

[6] Ferner RE, Aronson JK (2020). Chloroquine and hydroxychloroquine in covid-19. BMJ.

[7] Creech CB, Walker SC, Samuels RJ (2021). SARS-CoV-2 Vaccines. JAMA.

[8] Baden LR, El Sahly HM, Essink B, Kotloff K, Frey S, Novak R, Diemert D, Spector SA, Rouphael N, Creech CB (2021). Efficacy and Safety of the mRNA-1273 SARS-CoV-2 Vaccine. N Engl J Med.

[9] Thaker SK, Ch’ng J, Christofk HR (2019). Viral hijacking of cellular metabolism. BMC Biol.

[10] Fontaine KA, Sanchez EL, Camarda R, Lagunoff M (2015). Dengue virus induces and requires glycolysis for optimal replication. J Virol.

[11] Thaker SK, Chapa T, Garcia G, Gong D, Schmid EW, Arumugaswami V, Sun R, Christofk HR (2019). Differential metabolic reprogramming by zika virus promotes cell death in human versus mosquito cells. Cell Metab.

[12] Codo AC, Davanzo GG, Monteiro LB, de Souza GF, Muraro SP, Virgilio-da-Silva JV, Prodonoff JS, Carregari VC, de Biagi Junior CAO, Crunfli F (2020). Elevated Glucose Levels Favor SARS-CoV-2 Infection and Monocyte Response through a HIF-1alpha/Glycolysis-Dependent Axis. Cell Metab.

[13] Icard P, Lincet H, Wu Z, Coquerel A, Forgez P, Alifano M, Fournel L (2021). The key role of Warburg effect in SARS-CoV-2 replication and associated inflammatory response. Biochimie.

[14] Mullen PJ, Garcia G, Purkayastha A, Matulionis N, Schmid EW, Momcilovic M, Sen C, Langerman J, Ramaiah A, Shackelford DB (2021). SARS-CoV-2 infection rewires host cell metabolism and is potentially susceptible to mTORC1 inhibition. Nat Commun.

[15] Li S, Ma F, Yokota T, Garcia G, Palermo A, Wang Y, Farrell C, Wang YC, Wu R, Zhou Z (2021). Metabolic reprogramming and epigenetic changes of vital organs in SARS-CoV-2-induced systemic toxicity. JCI Insight.

[16] Korner RW, Majjouti M, Alcazar MAA, Mahabir E (2020). Of Mice and Men: The Coronavirus MHV and Mouse Models as a Translational Approach to Understand SARS-CoV-2. Viruses.

[17] Weiss SR (2020). Forty years with coronaviruses. J Exp Med.

[18] Homberger FR (1997). Enterotropic mouse hepatitis virus. Lab Anim.

[19] Roth-Cross JK, Bender SJ, Weiss SR (2008). Murine coronavirus mouse hepatitis virus is recognized by MDA5 and induces type I interferon in brain macrophages/microglia. J Virol.

[20] De Albuquerque N, Baig E, Ma X, Zhang J, He W, Rowe A, Habal M, Liu M, Shalev I, Downey GP (2006). Murine hepatitis virus strain 1 produces a clinically relevant model of severe acute respiratory syndrome in A/J mice. J Virol.

[21] Wang Y, Liu S, Liu H, Li W, Lin F, Jiang L, Li X, Xu P, Zhang L, Zhao L (2020). SARS-CoV-2 infection of the liver directly contributes to hepatic impairment in patients with COVID-19. J Hepatol.

[22] Schaefer IM, Padera RF, Solomon IH, Kanjilal S, Hammer MM, Hornick JL, Sholl LM (2020). In situ detection of SARS-CoV-2 in lungs and airways of patients with COVID-19. Mod Pathol.

[23] Lamers MM, Beumer J, van der Vaart J, Knoops K, Puschhof J, Breugem TI, Ravelli RBG, van PaulSchayck J, Mykytyn AZ, Duimel HQ (2020). SARS-CoV-2 productively infects human gut enterocytes. Science.

[24] Sariol A, Perlman S (2020). Lessons for COVID-19 Immunity from Other Coronavirus Infections. Immunity.

[25] Hirano N, Fujiwara K, Matumoto M (1976). Mouse hepatitis virus (MHV-2). Plaque assay and propagation in mouse cell line DBT cells. Jpn J Microbiol.

[26] Sanchez EL, Lagunoff M (2015). Viral activation of cellular metabolism. Virology.

[27] Moreno-Altamirano MMB, Kolstoe SE, Sanchez-Garcia FJ (2019). Virus control of cell metabolism for replication and evasion of host immune responses. Front Cell Infect Microbiol.

[28] Martinez-Reyes I, Chandel NS (2020). Mitochondrial TCA cycle metabolites control physiology and disease. Nat Commun.

[29] Horton RW, Meldrum BS, Bachelard HS (1973). Enzymic and cerebral metabolic effects of 2-deoxy-D-glucose. J Neurochem.

[30] Feksa LR, Cornelio AR, Dutra-Filho CS, de Souza Wyse AT, Wajner M, Wannmacher CM (2003). Characterization of the inhibition of pyruvate kinase caused by phenylalanine and phenylpyruvate in rat brain cortex. Brain Res.

[31] Bojkova D, Klann K, Koch B, Widera M, Krause D, Ciesek S, Cinatl J, Munch C (2020). Proteomics of SARS-CoV-2-infected host cells reveals therapy targets. Nature.

[32] Yang Y, Sauve AA (2016). NAD(+) metabolism: Bioenergetics, signaling and manipulation for therapy. Biochim Biophys Acta.

[33] Elhassan YS, Kluckova K, Fletcher RS, Schmidt MS, Garten A, Doig CL, Cartwright DM, Oakey L, Burley CV, Jenkinson N (2019). Nicotinamide riboside augments the aged human skeletal muscle NAD(+) metabolome and induces transcriptomic and anti-inflammatory signatures. Cell Rep.

[34] Ou X, Liu Y, Lei X, Li P, Mi D, Ren L, Guo L, Guo R, Chen T, Hu J (2020). Characterization of spike glycoprotein of SARS-CoV-2 on virus entry and its immune cross-reactivity with SARS-CoV. Nat Commun.

[35] Grabherr S, Ludewig B, Pikor NB (2021). Insights into coronavirus immunity taught by the murine coronavirus. Eur J Immunol.

[36] Ahmed W, Bertsch PM, Bibby K, Haramoto E, Hewitt J, Huygens F, Gyawali P, Korajkic A, Riddell S, Sherchan SP (2020). Decay of SARS-CoV-2 and surrogate murine hepatitis virus RNA in untreated wastewater to inform application in wastewater-based epidemiology. Environ Res.

[37] Matthews AE, Weiss SR, Shlomchik MJ, Hannum LG, Gombold JL, Paterson Y (2001). Antibody is required for clearance of infectious murine hepatitis virus A59 from the central nervous system, but not the liver. J Immunol.

[38] Ramakrishna C, Bergmann CC, Atkinson R, Stohlman SA (2003). Control of central nervous system viral persistence by neutralizing antibody. J Virol.

[39] Taylor PC, Adams AC, Hufford MM, de la Torre I, Winthrop K, Gottlieb RL (2021). Neutralizing monoclonal antibodies for treatment of COVID-19. Nat Rev Immunol.

[40] Gibellini L, De Biasi S, Paolini A, Borella R, Boraldi F, Mattioli M, Lo Tartaro D, Fidanza L, Caro-Maldonado A, Meschiari M (2020). Altered bioenergetics and mitochondrial dysfunction of monocytes in patients with COVID-19 pneumonia. EMBO Mol Med.

[41] Ajaz S, McPhail MJ, Singh KK, Mujib S, Trovato FM, Napoli S, Agarwal K (2021). Mitochondrial metabolic manipulation by SARS-CoV-2 in peripheral blood mononuclear cells of patients with COVID-19. Am J Physiol Cell Physiol.

[42] Tay MZ, Poh CM, Renia L, MacAry PA, Ng LFP (2020). The trinity of COVID-19: immunity, inflammation and intervention. Nat Rev Immunol.

[43] Liu G, Summer R (2019). Cellular metabolism in lung health and disease. Annu Rev Physiol.

[44] Smeitink JA, Zeviani M, Turnbull DM, Jacobs HT (2006). Mitochondrial medicine: a metabolic perspective on the pathology of oxidative phosphorylation disorders. Cell Metab.

[45] Houten SM, Violante S, Ventura FV, Wanders RJ (2016). The biochemistry and physiology of mitochondrial fatty acid beta-oxidation and its genetic disorders. Annu Rev Physiol.

[46] Scalise M, Pochini L, Galluccio M, Console L, Indiveri C (2017). Glutamine Transport and Mitochondrial Metabolism in Cancer Cell Growth. Front Oncol.

[47] Martorana A, Gentile C, Lauria A (2020). In Silico Insights into the SARS CoV-2 Main Protease Suggest NADH endogenous defences in the control of the pandemic coronavirus infection. Viruses.

[48] Ullrich S, Nitsche C (2020). The SARS-CoV-2 main protease as drug target. Bioorg Med Chem Lett.

[49] Miller R, Wentzel AR, Richards GA (2020). COVID-19: NAD(+) deficiency may predispose the aged, obese and type2 diabetics to mortality through its effect on SIRT1 activity. Med Hypotheses.

[50] Omran HM, Almaliki MS (2020). Influence of NAD+ as an ageing-related immunomodulator on COVID 19 infection: A hypothesis. J Infect Public Health.

[51] Trammell SA, Schmidt MS, Weidemann BJ, Redpath P, Jaksch F, Dellinger RW, Li Z, Abel ED, Migaud ME, Brenner C (2016). Nicotinamide riboside is uniquely and orally bioavailable in mice and humans. Nat Commun.

[52] Cengiz M, Borku Uysal B, Ikitimur H, Ozcan E, Islamoglu MS, Aktepe E, Yavuzer H, Yavuzer S (2020). Effect of oral l-Glutamine supplementation on Covid-19 treatment. Clin Nutr Exp.

