# Supplementary material for: Activation of TCA cycle restrains virus-metabolic hijacking and viral replication in mouse hepatitis virus-infected cells
Source: Cell Biosci. 2022 Jan 18;12:7. doi: 10.1186/s13578-021-00740-z (PMC8764321; doi:10.1186/s13578-021-00740-z)
Supplement: Supplementary file 1 — Additional file 1: Figure S1. Suppressed mitochondrial energy metabolism by glycolysis inhibitors. Extracellular acidification rates and oxygen consumption rates were measured before and after oligomycin treatment. Rate changes were normalized relative to baseline. DBT cells were treated for 30 h as indicated; 2-DG (2-deoxyglucose, 1mM), PHA (phenylalanine, 0.5 mM). Values represent means ± SD. *, p < 0.05. One-way ANOVA followed by a tukey's multiple comparison test was performed. All experiments were repeated at least 3 times. Figure S2. Cell viability of DBT cells treated for 30 h as indicated; 2-DG (2-deoxyglucose, 1 mM), PHA (phenylalanine, 0.5 mM), Pyr (pyruvate, 0.5 mM), DCA (dichloroacetate, 20 mM), NR (nicotinamide riboside, 400 μM), Omy (oligomycin, 2 μM), Rot (rotenone, 1 μM), FCCP (30 μM), FA (fatty acids, 10 mM), Gln (glutamine, 8 mM). Values represent means ± SD. *, p < 0.05. One-way ANOVA followed by a tukey’s multiple comparison test was performed. All experiments were repeated at least 3 times. Figure S3. Gene expression of TCA cycle in Calu-3 cells treated for 24 h as indicated; FA (fatty acids, 10 mM), NR (200 μM). Rplp0 was used for an internal control. Values represent means ± SD. *, p < 0.05. Student’s t-test was performed. All experiments were repeated at least 3 times. Figure S4. Intracellular pH of DBT cells treated for 24 h as indicated; Replete (Rep; glucose 450 mg/dl, pyruvate 0.5 mM), undernourished (glucose 50 mg/dl, pyruvate 0 mM), 2-DG (2-deoxyglucose, 1 mM), PHA (phenylalanine, 0.5 mM), Pyr (pyruvate, 0.5 mM), DCA (dichloroacetate, 20 mM), Omy (oligomycin, 2 μM), Rot (rotenone, 1 μM), FCCP (30 μM), FA (fatty acids, 10 mM), Gln (glutamine, 8 mM). Values represent means ± SD. *, p < 0.05. Student’s t-test and one-way ANOVA followed by a tukey’s multiple comparison test were performed. All experiments were repeated at least 3 times. [file 13578_2021_740_MOESM1_ESM.docx]

**Figures**

**Activation of TCA cycle restrains virus-metabolic hijacking and viral replication in mouse hepatitis virus-infected cells**

**Sang R. Lee^1^, Jeong Yeon Roh^1^, Jihoon Ryu^1^, Hyun-Jin Shin^1*^, and Eui-Ju Hong^1*^**

**^1^**College of Veterinary Medicine, Chungnam National University, Daejeon 34134, Republic of Korea

**^*^**Address correspondence to:

Hyun-Jin Shin, DVM, PhD

College of Veterinary Medicine, Chungnam National University

99 Daehak-ro, Yuseong-gu, Daejeon 34134, Korea

Phone: +82-42-821-6760; Email: shin0089@cnu.ac.kr

Eui-Ju Hong, DVM, PhD

College of Veterinary Medicine, Chungnam National University

99 Daehak-ro, Yuseong-gu, Daejeon 34134, Korea

Phone: +82-42-821-6781; Fax: +82-42-821-8903; Email: [ejhong@cnu.ac.kr](mailto:ejhong@cnu.ac.kr)


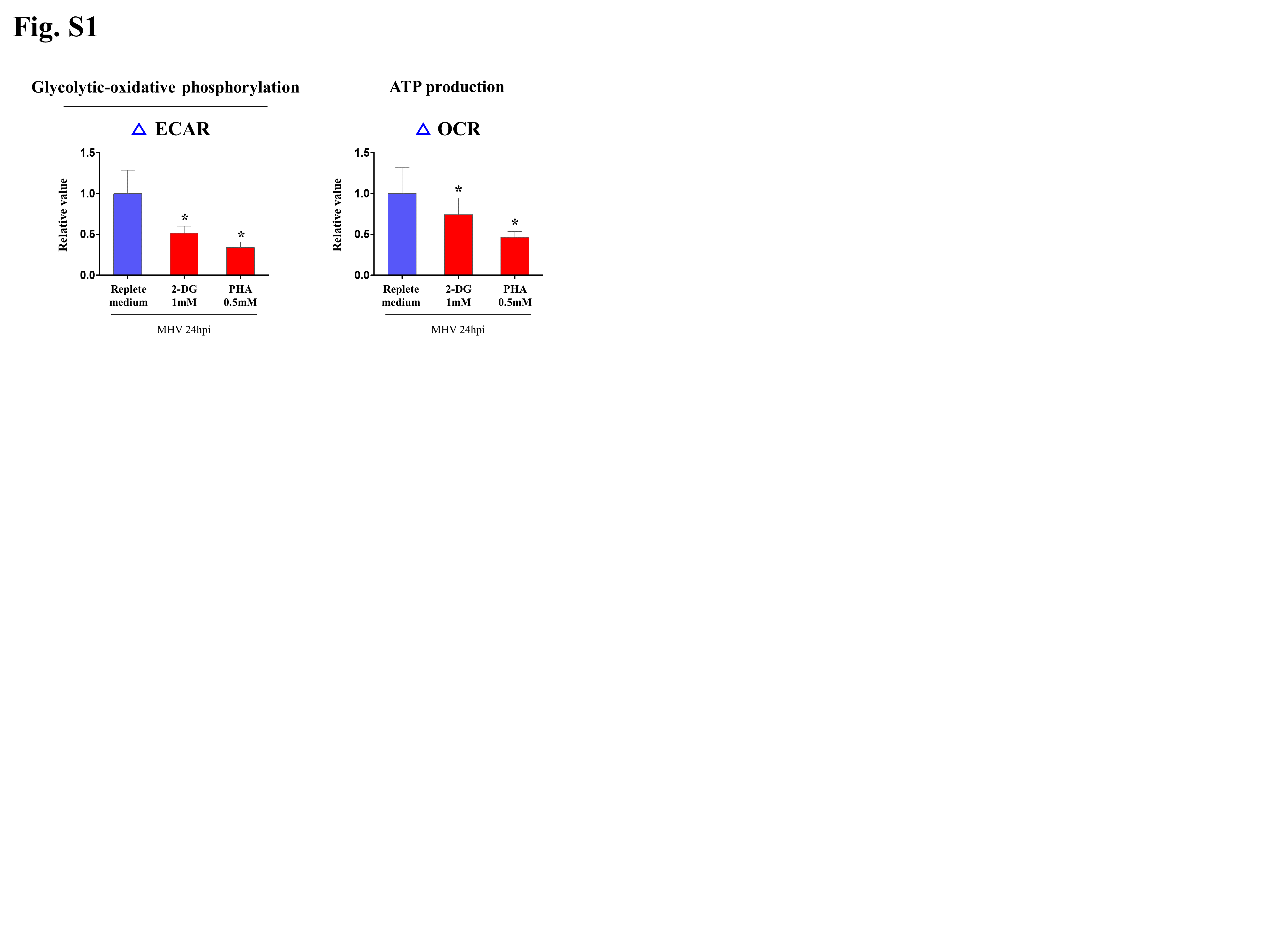


**Figure S1.** Suppressed mitochondrial energy metabolism by glycolysis inhibitors. Extracellular acidification rates and oxygen consumption rates were measured before and after oligomycin treatment. Rate changes were normalized relative to baseline. DBT cells were treated for 30 h as indicated; 2-DG (2-deoxyglucose, 1mM), PHA (phenylalanine, 0.5 mM). Values represent means ± SD. *, *p*<0.05. One-way ANOVA followed by a tukey's multiple comparison test was performed. All experiments were repeated at least 3 times.


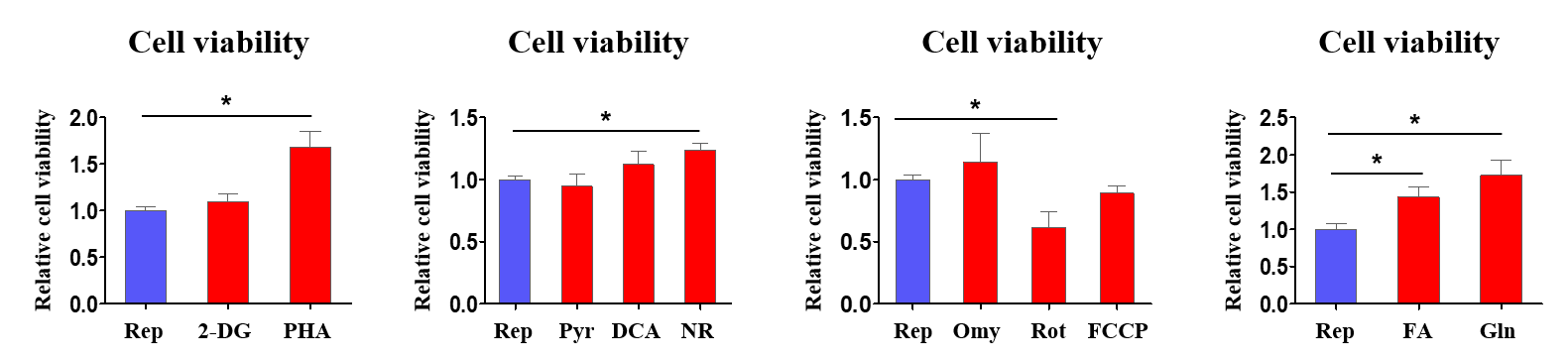


**Figure S2.** Cell viability of DBT cells treated for 30 h as indicated; 2-DG (2-deoxyglucose, 1mM), PHA (phenylalanine, 0.5 mM), Pyr (pyruvate, 0.5 mM), DCA (dichloroacetate, 20mM), NR (nicotinamide riboside, 400 μM), Omy (oligomycin, 2 μM), Rot (rotenone, 1 μM), FCCP (30 μM), FA (fatty acids, 10 mM), Gln (glutamine, 8 mM). Values represent means ± SD. *, p<0.05. One-way ANOVA followed by a tukey's multiple comparison test was performed. All experiments were repeated at least 3 times.


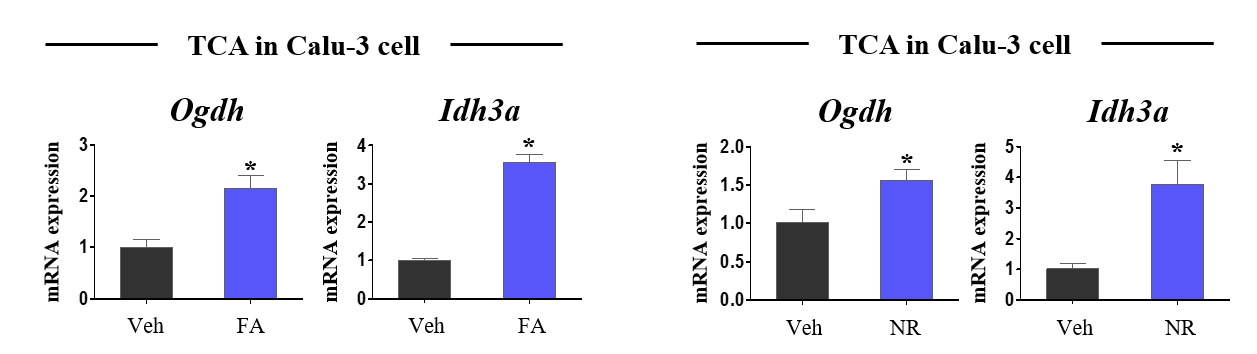


**Figure S3.** Gene expression of TCA cycle in Calu-3 cells treated for 24 h as indicated; FA (fatty acids, 10mM), NR (200 μM). *Rplp0* was used for an internal control. Values represent means ± SD. *, p<0.05. Student’s *t*-test was performed. All experiments were repeated at least 3 times.


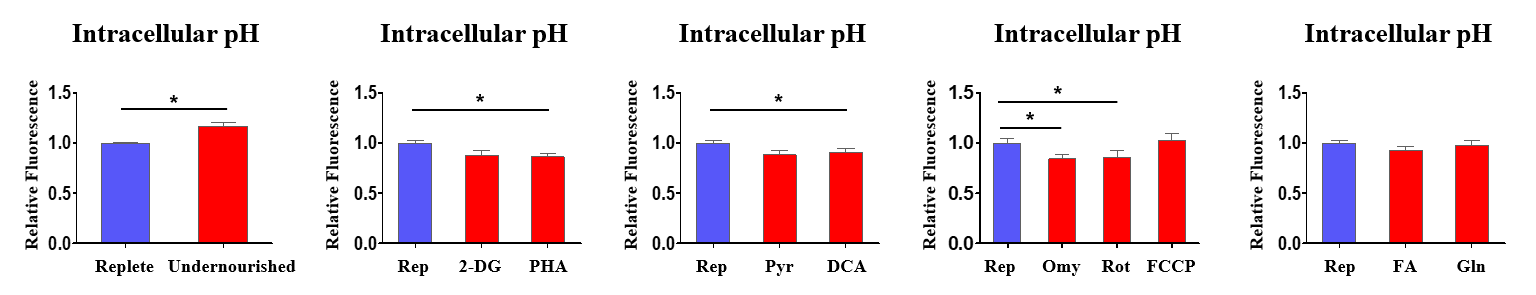


**Figure S4.** Intracellular pH of DBT cells treated for 24 h as indicated; Replete (Rep; glucose 450 mg/dl, pyruvate 0.5 mM), undernourished (glucose 50 mg/dl, pyruvate 0 mM), 2-DG (2-deoxyglucose, 1mM), PHA (phenylalanine, 0.5mM), Pyr (pyruvate, 0.5mM), DCA (dichloroacetate, 20mM), Omy (oligomycin, 2μM), Rot (rotenone, 1μM), FCCP (30μM), FA (fatty acids, 10mM), Gln (glutamine, 8mM). Values represent means ± SD. *, p<0.05. Student’s *t*-test and one-way ANOVA followed by a tukey's multiple comparison test were performed. All experiments were repeated at least 3 times.
